# The impact of tucking on fertility among transgender women: A systematic review

**DOI:** 10.1080/26895269.2024.2387643

**Published:** 2024-08-07

**Authors:** Ethan Manafi, André R. Belarmino, Jesse N. Mills, Sriram V. Eleswarapu, Gladys Y. Ng

**Affiliations:** a Division of Andrology, Department of Urology, David Geffen School of Medicine, University of California, Los Angeles, Los Angeles, CA, USA; b UCLA Gender Health Program, David Geffen School of Medicine, University of California, Los Angeles, Los Angeles, CA, USA

**Keywords:** transgender persons, semen analysis, genital tucking, fertility preservation, infertility

## Abstract

**Introduction:**

Tucking involves maneuvering the testicles into the inguinal canal and compressing one’s penis and scrotum posteriorly for a smoother pelvic area. A study based in Baltimore reports that 74.7% of transgender women engage in this practice.

**Objectives:**

Fertility preservation is a significant concern for many transgender women, however, the implications of tucking on fertility remain underexplored. This review aims to bridge this knowledge gap.

**Methods:**

Using PRIMSA guidelines, a systematic search across various databases was conducted to identify studies evaluating the impact of genital tucking on fertility. Keywords related to tucking, transgender identity, and fertility were utilized.

**Results:**

In total, 127 manuscripts were identified, of which 11 satisfied the inclusion and exclusion criteria. Four studies noted significant differences in semen parameters between cisgender men and transgender women prior to gender-affirming hormone therapy suggesting tucking as a potential factor. Two studies documented tucking prior to semen analysis but did not establish a definitive link between tucking and lower semen parameters in transgender women. One study, with a larger sample size of 113 transgender women, discovered an odds ratio of 7.95 between extensive tucking and low total motile sperm count. One year-long study on daily tucking reported a decline of up to 98% in total motile sperm count, and a complementary paper noted an increase in abnormal sperm morphology. Two case reports observed that after a 3–4 month cessation from tucking, semen parameters returned to normal.

**Conclusion:**

The review highlights the prevalence of tucking among transgender women and the negative impact on fertility it may have. Temporary cessation of tucking may improve semen parameters for fertility preservation. A harm reduction approach should be implemented to balance fertility aspirations with the management of gender dysphoria.

## Introduction

According to the Williams Institute at UCLA School of Law, approximately 1.6 million Americans aged 13 and older identify as transgender, with 18% of this population being adolescents between the ages of 13 and 17 (Herman et al., 2022). Continued research and clinical focus on pre­ventative medicine and screening needs of transgender individuals is crucial to ensure comprehensive and inclusive healthcare for this growing population.

Some transgender individuals seek gender-affirming hormone therapy (GAHT) and surgeries to mitigate their dysphoria or achieve social-gender congruence. Beyond medical interventions, many adopt specific gender-affirming behaviors like ‘tucking’ to modify their physique in accordance with their gender. Tucking involves maneuvering the testicles into the inguinal canal and the penis and scrotum posteriorly between the legs with the purpose of achieving a visibly flat and smooth appearance in the pelvic area (Coleman et al., 2022; Deutsch, 2016). To maintain this alignment, individuals might opt for snug underwear, use a specialized underwear known as a gaff, or apply duct tape to ensure the tuck remains secure; though individual practices can vary widely. This practice enables some transgender women to align more closely with their gender and alleviate gender dysphoria. It can also allow some to maintain discretion about their identity in social scenarios and enhance personal safety to avoid transphobic violence.

According to Malik et al. (2024), 74.7% (*N* = 59/79) of transgender women in Baltimore reported engaging in tucking, and utilized a variety of methods such as gaffs, tight panties, and duct tape. Among those who tuck, 84.5% tucked daily, with 44.8% tucking for 17 or more hours each day. Additionally, 67.2% had been tucking for over seven years. de Nie et al. (2022) studied 113 transgender women in Amsterdam and found that, by broadly defining tucking as both positioning the testicles or wearing tight underwear for concealment, similar to Malik’s definition, 72.6% of participants practiced some form of tucking. A survey conducted by Kidd et al. (2024) in Virginia (*N* = 98) reported that 79% of patients with gender dysphoria assigned male at birth practice tucking, albeit at varying frequencies. Of the total respondents, 51% tuck for a duration of 8 to 13 h per day, and 43% began tucking at 18 or younger. Additionally, half of the participants in Malik et al. expressed concerns about potential health-related outcomes of tucking, underscoring the importance of research in this area.

The implications of tucking on fertility are currently under-researched. A study conducted in Germany found that 76.1% of transgender women have considered fertility preservation, and two-thirds reported a desire for children, highlighting that fertility could hold significance for some transgender women (Auer et al., 2018). Gender-affirming surgeries, such as orchiectomy or vaginoplasty, lead to irreversible sterilization, while GAHT adversely affects fertility as well (Adeleye et al., 2019; Schneider et al., 2017; Vereecke et al., 2021). Patients may want to explore cryopreservation options; however, tucking may compromise their fertility potential even before they entertain the idea of fertility preservation.

Thermal regulation plays an essential role in sperm health and tucking exposes testicles to higher body temperatures. Spermatogenesis, the production of sperm, thrives in an environment that is a few degrees cooler than the body’s core temperature (Kim et al., 2013; Neto et al., 2016). However, tucking can disrupt this thermal equilibrium, leading to testicular hyperthermia and diminished sperm quality. Past studies on cisgender men have revealed that tighter underwear, which raises scrotal temperature, can lower sperm count, concentration, motility, and morphology (Kaya et al., 2020; Mínguez-Alarcón et al., 2018; Tiemessen et al., 1996). Similarly, tucking could impair sperm health by increasing the temperature and disrupting spermatogenesis. However, the relationship between tucking and fertility remains a critical knowledge gap.

Existing guidelines on transgender care consistently caution about the uncertainty surrounding tucking’s effects on fertility, urging exploration into the potential consequences of tucking and emphasizing the need for further research (Coleman et al., 2022). Through this systematic review, we aim to clarify the impact of tucking on fertility, providing a foundation for informed clinical decisions, patient counseling, and future research.

## Material and methods

In accordance with PRISMA guidelines we performed a comprehensive systematic search across several databases, including PubMed Central, PubMed, Web of Science, and Medline. Keywords included “tucking,” “genital concealment,” “tight underwear,” “transgender,” “gender dysphoria,” “fertility,” and “semen quality.”

We identified 127 publications. Of those, 123 were identified using databases − 97 from PubMed Central, 9 from PubMed, 11 from Web of Science, and 6 from Medline, and 4 papers were identified by microsearching the reference lists for relevant papers. Databases were searched from inception through September 2023. The titles and abstracts of each result were monitored to identify relevant papers. Articles addressing unrelated topics, such as fertility issues in cisgender men not associated with tucking, reproductive care for cisgender women, descriptions of cryopreservation methods, assisted reproductive technologies, historical or political analyses of transgender care, nonpeer-reviewed theses, and general transgender care papers that merely introduced or provided a basic definition of tucking without offering empirical data or in-depth analysis, were excluded. Duplicates were also removed.

**Figure 1.**
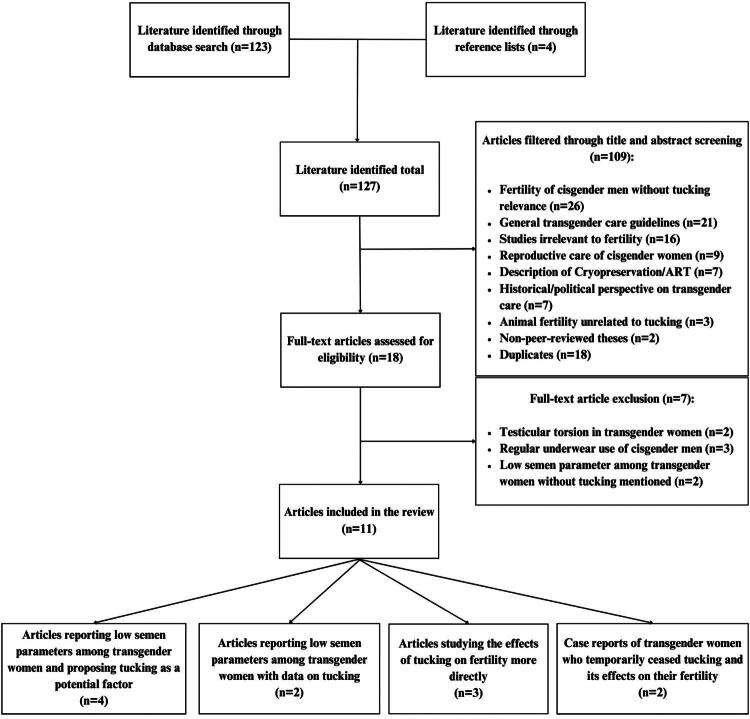
Flow chart illustrating the inclusion and exclusion criteria in the study.

Remaining studies (*n* = 18) were read in full text to determine their eligibility. Two researchers read through each of the selected papers to extract relevant information, including study design, sample size, comparison groups, semen parameters, presence and details of tucking practices, impact of cessation from tucking on semen quality, and statistical analyses. Following the full-text review, we included 11 studies that directly referenced or detailed the act of tucking in the context of fertility. Papers that did not directly address the objectives of this review were excluded, including studies mentioning testicular torsion in transgender women (*n* = 2) (Debarbo, 2020; Epps et al., 2016), studies highlighting the effects of cisgender men’s regular underwear use on fertility (*n* = 3) (Kaya et al., 2020; Mínguez-Alarcón et al., 2018; Tiemessen et al., 1996), and studies reporting low semen parameters among transgender women without mention of tucking (*n* = 2) (Hamada et al., 2015; Li et al., 2018). A total of 11 studies remained for this review, and key findings from each study were then synthesized. A schematic of the workflow for selecting manuscripts for review is detailed in Figure 1.

## Results

A summary of the studies included in this review is presented in Table 1.

**Table 1.** Studies included in this review.

Studies	Study design	Country	Sample size	Comparison group	Condition studied	Tucking	Relevant findings
de Nie et al. (2020)	Retrospective cohort (1972–2017)	Netherlands	260 transgender women	General population of unscreened cisgender men (WHO data)	Semen quality pre-GAHT	Tucking proposed as a potential factor affecting semen quality, but not specifically measured	Transgender women pre-GAHT have significantly lower semen parameters and increased abnormalities compared to the general population of cisgender men. They also have low TMSC post-thaw.
Rodriguez-Wallberg et al. (2021)	Prospective cohort (2013–2018)	Sweden	177 transgender women	General population of unscreened cisgender men (WHO data)	Semen quality in patients pre-GAHT and those post-GAHT	Tucking proposed as a potential factor affecting semen quality, but not specifically measured	Transgender women pre- and post-GAHT have significantly lower semen quality compared to the general population of cisgender men, with a more pronounced disparity between those with a history of GAHT.
Sermondade et al. (2021)	Retrospective cohort (2018–2020)	France	82 transgender women	Cisgender men sperm donors with normal sperm parameters (2018–2020)	Semen quality in patients pre-GAHT and those post-GAHT	Tucking proposed as a potential factor affecting semen quality, but not specifically measured	Patients pre-GAHT showed decreased sperm morphology, with cases of teratozoospermia and azoospermia compared to sperm donors; other parameters were not significantly different. Patients post-GAHT exhibited more significant diminished semen parameters and increased abnormalities.
Barda et al. (2023)	Retrospective cohort (2000–2019)	Israel	79 transgender women	Self-reported healthy cisgender male sperm donors	Semen quality in patients pre-GAHT and those with prior history of GAHT	Tucking proposed as a potential factor affecting semen quality, but not specifically measured	Transgender women pre-GAHT and with prior history of GAHT have significantly lower semen quality compared to the general population of cisgender men, with a more pronounced disparity between those with a prior history of GAHT.
Marsh et al. (2019)	Case-control	USA	22 transgender women	17 fertile cisgender men who fathered a child within the last 24 months	Semen quality pre-GAHT	Tucking was recorded; however, without any details of frequency or duration. *N* = 7 for subjects who tucked	Transgender women pre-GAHT exhibited significantly lower semen quality than fertile cisgender men. Weak to very weak correlations observed between tucking and low semen parameters.
Amir et al. (2022)	Retrospective cohort (2013–2020)	Israel	26 adolescent transgender women	General population of unscreened cisgender men (WHO data)	Semen quality pre-GAHT	Tucking was recorded; however, without any details of frequency or duration. *N* = 7 for subjects who tucked.	Transgender women pre-GAHT exhibited significantly higher sperm abnormalities, low semen parameters, and diminished post-thaw TMSC compared to cisgender men. No significant effect of tucking on low semen quality was found.
de Nie et al. (2022)	Prospective cohort (2018–2020)	Netherlands	113 transgender women	General population of unscreened cisgender men (WHO data)	Semen quality pre-GAHT	Data were recorded on wearing tight underwear and tucking, including the frequency (times per month) and duration (hours per day)	Semen quality of transgender women pre-GAHT was significantly lower than the general population of cisgender men across every parameter. A TMSC of <5 million was associated with always wearing tight underwear (odds ratio: 2.89) and tucking more than 8 times a month (odds ratio: 7.95), after correcting for confounding variables.
Mieusset et al. (1985)	Experimental study	France	14 cisgender men	Subjects served as their own baseline comparison	Semen quality during tucking as a potential method for male contraception	Instructed cisgender men to tuck daily during waking hours using specialized underwear for one year	All sperm characteristics declined significantly after the initial weeks, reaching their lowest levels between 28 and 52 wk. Values returned to baseline levels within 6–8 months of tucking cessation.
Mieusset et al. (1987)	Experimental study	France	19 cisgender men	Subjects served as their own baseline comparison	Sperm morphology during tucking as a potential method for male contraception	Instructed cisgender men to tuck daily during waking hours using specialized underwear for at least 6 months	Sperm parameters declined, and abnormally shaped spermatozoa increased within the second month of the experiment. Normal morphology returned to baseline levels within 8 months of tucking cessation.
Trussler and Carrasquillo (2020)	Case report	USA	1 transgender woman	Subject served as her own baseline comparison	Semen parameters after a pause from tucking	Patient regularly engaged in tucking	In a patient experiencing cryptozoospermia and negligible sperm count, semen parameters returned to near-normal levels after a 3-month pause from tucking.
Turley and Potdar (2023)	Case report	UK	1 transgender woman	Subject served as her own baseline comparison	Semen parameters after a pause from tucking	Patient regularly engaged in tucking	In a patient with severely poor semen quality, sperm count and motility returned to normal levels after a 3–4 month pause from tucking. Morphology improved but did not return to normal levels.

Several studies reported differences in semen parameters between cisgender men and transgender women prior to starting GAHT and discussed tucking as a potential factor. de Nie et al. (2020) analyzed semen quality of 260 transgender women from 1972 to 2017 in the Netherlands. Compared to the World Health Organization (WHO)’s data on semen analysis from the general population, transgender women exhibited lower semen volume (2.7 vs. 3.2 mL), lower sperm concentration (40 vs. 64 million/mL), lower total sperm count (103 vs. 196 million), and reduced progressive motility (41% vs. 57%). Among the subjects, 8.1% had azoospermia, 18.1% displayed oligoasthenozoospermia, 9.6% had oligozoospermia, and 10.8% had asthenozoospermia. Samples were cryopreserved and after thawing, the total motile sperm count (TMSC) amongst the transgender individuals’ samples averaged 1.0 million per vial. Notably, 73.6% of the samples were deemed suitable only for advanced assisted reproductive technologies (ART), such as *in vitro* fertilization (IVF) or intracytoplasmic sperm injection (ICSI). After controlling for common contributors to reduced semen quality, like alcohol, cannabis use, hormonal treatments, BMI, and depression, the authors proposed tucking as a potential factor for the observed discrepancies.

Rodriguez-Wallberg et al. (2021) examined semen health parameters in transgender women in relation to GAHT in Stockholm. In a prospective cohort study spanning 2013 to 2018, data from 177 participants were analyzed. Transgender women pre-GAHT demonstrated a decline in semen characteristics when compared to the population of unscreened cisgender men, using data from WHO. Specifically, among transgender women pre-GAHT, the prevalence of low sperm concentration was 24.2%, low total sperm count was 21.7%, and impaired sperm motility was 16%. In contrast, only 5% of cisgender men exhibited impairments in each of these categories. Tucking was suggested as a potential factor influencing these outcomes.

Sermondade et al. (2021) conducted a retrospective study from 2018 to 2020 in France. Compared to cisgender men sperm donors, the study found higher cases of teratozoospermia and azoospermia among the 65 transgender women with no prior history of GAHT. Barda et al. (2023) conducted a retrospective analysis of 79 transgender women spanning from 2000 to 2019. The results revealed significant differences in fresh semen analysis between sperm donors and transgender women, with pronounced disparities in total sperm count, motility, and total motile sperm count, and azoospermia. Notably, these disparities in semen parameters widened in post-thaw samples, with 77.9% of samples from transgender women being suitable only for IVF or ICSI. Both studies discussed tucking as a factor that could contribute to these findings.

Two studies specifically documented the practice of tucking while evaluating semen quality in transgender women. Marsh et al. (2019) compared the semen quality of adult transgender women to fertile cisgender men in a case-control study. The transgender cohort, consisting of 22 individuals revealed a concentration of 31.89 million/mL compared to 54.6 million/mL, total sperm per ejaculation of 44.49 million compared to 183.1 million, TMSC of 27.63 million compared to 106.6 million, a volume of 2.4 mL compared to 3.6 mL, and Kruger strict morphology of 3.0% (impaired) versus 4.5%. Amir et al. (2022) evaluated semen differences between adolescent transgender women and cisgender males. The study unveiled a series of abnormalities in the transgender cohort including 72% teratozoospermia, 28% oligozoospermia, and 4% azoospermia. Other differences included volume (1.46 vs. 3.2 mL), concentration (28 vs. 64 million/mL), total sperm count (28.2 vs. 196 million), total motility (51.6% vs. 62%), and normal Kruger strict morphology (2% vs. 14%). Post-thaw results in the transgender group revealed a TMSC average of 0.17 million, with 87.7% of samples suitable only for ICSI. Both studies attempted to correlate their findings with tucking. Marsh et al. found only weak to very weak correlations between tucking and semen parameters, and Amir et al. found no significant effect. However, both studies noted limitations due to small sample sizes of participants who tucked (*N* = 7 in both studies) and lack of data on individual tucking habits, including frequency and duration.

We identified three studies that directly assessed the impact of tucking on semen parameters.

de Nie et al. (2022) conducted a cohort study examining the impact of specific behaviors, such as wearing tight underwear and tucking, on semen quality in transgender women prior to initiating hormonal therapy. The study, involving 113 participants, revealed worse semen parameters in transgender women compared to the general population across all parameters. Wearing tight underwear consistently was associated with a TMSC of <5 million, with odds ratios of 3.06 and then 2.89 after adjusting for potential confounding factors, such as age, BMI, or lifestyle factors. Additionally, tucking more than 8 times a month exhibited a stronger correlation with a diminished TMSC, with odds ratios of 7.95 after correcting for confounding variables. In 1985, Mieusset and colleagues investigated testicular thermogenesis as a potential avenue for male contraceptives. Over a year-long study 14 cisgender men were instructed to tuck by keeping their testicles in the inguinal canal during waking hours using specialized underwear. Throughout the study, all sperm characteristics declined significantly after the initial weeks, reaching their lowest levels between 28 and 52 wk. Total sperm count toward the end of their experiment revealed 12–34 million/ejaculate, down from an initial 255 ± 135 million/ejaculate prior to tucking; sperm concentration declined to 3–10 million/mL, from 72 ± 30 million/mL; motility dropped to 21–34%, from 68%±7%; and the total motile sperm count was between 4–12 million/ejaculation, down from 174 ± 89 million/ejaculation, a decline of 92–98%. Notably, sperm characteristics began to rebound within a month of discontinuing the tucking practice, and values returned to baseline levels within 6–8 months. In a complementary publication, Mieusset et al. (1987) reported on sperm morphology. They observed the rate of abnormally shaped spermatozoa increased from 30% to 50% after the second month of daily tucking and maintained similar values until the end of the study.

Lastly we identified two case reports that shed direct insight into the impact of tucking and the duration of cessation that may yield a return to baseline semen analysis characteristics. Trussler and Carrasquillo (2020) and Turley and Potdar (2023) both highlighted individual cases of two transgender women who regularly practiced tucking and later sought fertility preservation. In both instances, the initial semen analyses revealed significant abnormalities: Trussler and Carrasquillo reported a case with cryptozoospermia, while Turley and Potdar described a patient exhibiting severe oligoasthenoteratozoospermia. Both patients ceased tucking temporarily, leading to significant improvements in their semen parameters. The patient in the 2020 report saw her total sperm count increase from almost negligible to 182.8 million with enhanced sperm morphology after a 3-month break from tucking. Similarly, the patient in the 2023 report witnessed her sperm count rise from a mere 3 sperm cells post-centrifugation to 72 million/mL in 3 months and further to 116 million/mL after 4 months. Sperm motility too showed marked improvement, and while there was an increase in normal morphology, teratozoospermia persisted in this case. Trussler and Carrasquillo ruled out hormonal, genetic, or obstructive factors as potential contributors. Both studies attributed the initial reduced sperm quality to the proximity of the testes to the abdomen, and testicular heat stress during tucking, believed to disrupt spermatogenesis.

## Discussion

This study represents the first systematic review examining the impacts of genital tucking on fertility among transgender women. The findings suggest that genital tucking likely negatively impacts semen parameters and fertility. This impact may vary by factors such as frequency, duration, methods, or devices used for tucking, as well as the time since the individual started to perform tucking. Transgender women, even before initiating GAHT, have significantly impaired sperm characteristics compared to cisgender men and demonstrate a variety of semen abnormalities. Notably, de Nie et al. (2022) reported that individuals who tuck more than 8 times a month have an almost eightfold higher likelihood of exhibiting TMSC of less than 5 million, and Mieusset et al. (1985) reported a decline of up to 98% in TMSC after a year of daily tucking. Abstaining from tucking for periods of 3–4 months may enhance semen quality and facilitate successful fertility preservation prior to initiating further gender-affirming medical interventions known to compromise fertility, such as orchiectomy.

These insights on tucking can help optimize the fertility preservation process for transgender patients. The execution of a successful fertility preservation process is crucial to transgender women for several reasons, including the desire for biological children, and minimizing delays in initiating GAHT (Bayar et al., 2023). Additionally, by addressing fertility issues caused by tucking, the financial burden of ART may be lowered. Our review suggests that the majority of patients were constrained to utilizing expensive, more advanced ART methods such as IVF and ICSI owing to their suboptimal sperm. This is particularly salient as transgender individuals frequently encounter financial stress due to discrimination, lack of job protection, familial estrangement, and housing precarity (Grant et al., 2011; James et al., 2024). Improved sperm quality may enable the use of intrauterine insemination (IUI), which is less costly and more widely available (Marcus et al., 2011).

It is important to note that providers may be wise to adopt a harm-reduction model when approaching tucking. Transgender women already face elevated stressors and mental health distress due to stigma and discrimination, and tucking could ease the dysphoria experienced by patients (Lin et al., 2021; Liu et al., 2024; Matouk et al., 2023). Providers should balance the risk of gender dysphoria with fertility considerations in the context of family planning aspirations and foster comprehensive counseling before fertility preservation. They should share their knowledge on this subject and champion informed decision-making among their patients regarding gender-affirming care, fertility preservation, and tucking practices. Additionally, transgender individuals often delay doctor visits and avoid checkups due to trauma within the healthcare system and fear of discrimination (Seelman et al., 2017). Collaborative initiatives must be established with community organizers to spread information on the potential effects of tucking on fertility, providing clear evidence and approaches to mitigate the fertility risks.

The landscape of gender nuances and identities is undergoing dynamic shifts. The findings of this review could resonate with individuals who tuck but may not identify as transgender women, such as nonbinary people, drag queens, or other queer individuals. Hence, it is imperative for all healthcare providers, and not just those who provide care to transgender patients specifically, to be well-versed on tucking and its implications.

Limitations of this review include a paucity of relevant studies, resulting in a relatively small number of included articles. Out of the seven cohort and case-control studies reporting on poor sperm quality among transgender women, only three presented data on tucking practices, two of which had only seven participants who tuck. de Nie et al. (2022) conducted the sole cohort study with a larger sample size, reporting a strong correlation between tucking and sperm health. Mieusset et al. (1985, 1987) also demonstrated robust evidence for tucking negatively affecting sperm parameters, but this was part of an experimental study on cisgender men and may not be representative of the real-life practices of transgender women. Additionally, the diversity in the ways individuals engage in tucking and the various methods employed presents challenges in comparing data.

There is substantial need for further research in this area. One pressing need is for the establishment of evidence-based guidelines on tucking practices. Such guidelines could provide safer techniques that minimize the impact on fertility while still addressing dysphoria. Future studies should aim to compare various methods and techniques used by transgender women to tuck, such as gaffes, tight underwear, duct tape and identify their different impacts on fertility. They should also explore tucking cessation timelines, or possibility of intermittent tucking, prior to fertility preservation to balance optimal semen analysis with management of gender dysphoria. Lastly, researchers could examine correlations between race, socioeconomic status, or geographical location and the use of specific tucking practices or devices and determine the socio-cultural aspects influencing individual decisions. All of these will help to formulate a safe and holistic tucking guideline for patients who wish to have biological children and inform how providers could best consult them prior to fertility preservation.

## Conclusion

The reviewed studies highlight the prevalence of tucking among transgender women and the negative impact on fertility it may have. Temporary cessation of tucking may improve semen parameters for fertility preservation. A harm reduction approach should be implemented to balance fertility aspirations with the management of gender dysphoria. Future research should explore the impact of various tucking methods on fertility and investigate the optimal timelines for a temporary pause prior to fertility preservation to formulate safe and holistic guidelines for providing care to transgender individuals.
